# Preparation and evaluation of novel functional fermented dairy products containing propolis and cinnamon

**DOI:** 10.1007/s13197-021-05255-6

**Published:** 2021-10-03

**Authors:** Ayşe Gunes-Bayir, Mehmet Gültekin Bilgin, Duygu Guclu, Sultan Pogda, Agnes Dadak

**Affiliations:** 1 Department of Nutrition and Dietetics, Faculty of Health Sciences, Bezmialem Vakif University, Silahtarağa Caddesi No: 198, Eyüpsultan, 34065 Istanbul, Turkey; 2 Department of Nutrition and Dietetics, Faculty of Health Sciences, Bezmialem Vakif University, Eyüpsultan, 34065 Istanbul, Turkey; 3 Res. Assist, Department of Nutrition and Dietetics, Faculty of Health Sciences, Bezmialem Vakif University, Eyüpsultan, 34065 Istanbul, Turkey; 4 Dietitian, Istanbul, Turkey; 5 Institute of Pharmacology, Department for Biomedical Sciences, University of Veterinary Medicine Vienna, Veterinärplatz 1, 1210 Vienna, Austria

**Keywords:** Probiotic yogurt, Cinnamon, Propolis, Bacteria, SARS-CoV-2 infection

## Abstract

Novel functional food products might be an easy accessible and eligible approach to help reduce the risk of severe viral infections including SARS-CoV-2. Hence a product containing probiotics, propolis and cinnamon was developed and interferences of the ingredients were characterized. Yogurts were prepared using starter cultures with propolis (0.03%) and cinnamon in various concentrations (0.3%, 1%, and 2.5%). *Bifidobacterium animalis* ssp. *lactis*, *Lactobacillus acidophilus, Streptococcus thermophilus*, and *Lactobacillus delbrueckii* subsp. *bulgaricus* were used as microorganisms for yogurt production. Chemical analysis revealed a decline of fat matter in the presence of propolis and/or cinnamon. Propolis had statistically significant suppressive effects on *Bifidobacterium animalis* ssp. *lactis* as well as on *Lactobacillus delbrueckii* subsp. *bulgaricus* numbers (*p* < 0.05). These effects were diminished in the presence of increasing cinnamon concentrations. For *Lactobacillus acidophilus* a statistically significant reducing effect on the number of colonies was observed in all products investigated. Nevertheless all samples met the standard of recommended level of ≥ 10^6^ viable cells/g of a product. Propolis showed an inverse effect on *Streptococcus thermophilus* by increasing its colony numbers in yogurts. The probiotic yogurt samples containing propolis (0.03%) and cinnamon (2.5%) gained the highest number of points in the sensory evaluation compared to control.

## Introduction

Propolis has gained strong scientific interest during the current COVID-19 pandemic since it has not only proven antiinflammatory and immunoregulatory effects but also potent antiviral activity against pathogens that cause severe syndromes, including those caused by coronaviruses (Berretta et al. 2020; Lima et al. 2020). Among other effects, propolis has been shown to inhibit the attachment of SARS-CoV-2 to Angiotensin-converting enzyme 2 (ACE2), a major target of the virus for host cell invasion. Beretta et al. (2020) emphasized that propolis does not interact with the main liver enzymes or with other key enzymes and therefore can be used concurrently with the main drugs without risk of potentiation or inactivation. Moreover, propolis has been shown to stimulate the adaptive immune response promoting prophylactic antiviral effects (Babaei et al. 2016). It has also been suggested that several essential oils express activity against SARS-CoV-2 virus. Kulkarni et al. (2020) evaluated the efficacy of various essential oils among others obtained from *Cinnamomum zeylanicum* to block the receptor binding domain (S1) subunit of spike (S) proteins of SARS-CoV-2. S1 protein is involved in the interaction with ACE2 receptors. The *in-silico* study revealed that cinnamaldehyde and cinnamyl acetate showed potential to inhibit S1 subunit of S proteins (Kulkarni et al. 2020). Since SARS-CoV-2 infection is associated with immune dysfunction and gut microbiota alterations, probiotics are recently as well under discussion as a preventive approach due to their positive impact on the microbiota composition and host immune functions (Hu et al. 2021; Singh and Rao 2021).

In recent years, the importance of functional foods on human health has been increasingly recognized (Nigam 2018). Probiotics have gained reputation as healthy components of functional foods. In different cultures, prevention and therapy against viruses are traditionally based on the combination of several functional foods and nutraceuticals with active immunomodulators, polyphenols, antiinflammatory and antioxidation components (Alkhatib 2020). However, less is known about the role of functional foods in viral infections such as SARS-CoV-2. Probiotic cultures are defined as single or mixed cultures that express beneficial effects on human and animal intestines, which prevent the growth of pathogens and strengthen the immune systems (Sazawal et al. 2006). Probiotics are known to have antimicrobial properties and to be able to suppress various pathogens such as *Escherichia coli*, *Staphylococcus aureus*, *Shigella sonnei*, *Shigella flexneri*, *Campylobacter jejuni* and *Salmonella typhimurium* (Tavakoli et al. 2019). According to the Turkish Food Codex at least 1.0 × 10^6^ colony forming unit (cfu)/g live probiotic microorganisms must be contained in probiotic foods (Turkish Food Codex, 2017). Yogurt is considered as the most important probiotic source among the fermented milk products and ranks first in the list of preferred products due to many beneficial effects (Sarkar 2019). Studies on daily yogurt intake reported that yogurt has positive effects on cellular immune function and oxidative stress (Irvine 2011). Yogurt applies positive bioactive effects through the high nutritional value including its abundance of calcium, zinc, and vitamin B (Lourens-Hattingh and Viljoen, 2011). This nutritional value can be enhanced by adding probiotic bacteria to the yogurt since probiotic bacteria help to improve the intestinal microflora (Irvine et al. 2011). As outlined above, recent COVID-19 research suggests intake of probiotics as well as propolis and cinnamon intake on a regular basis as being potentially beneficial in helping to prevent SARS-CoV-2 infections. To our knowledge, no data are available on the effects of combined intake of yogurt, propolis and cinnamon or its active ingredients. In a study performed in rats, propolis mixed into yogurt induced hypoglycemic effects with reduction of serum levels of cholesterol (Bukhari et al. 2012). Adding cinnamon, which contains mucilage, sugar, resin, tannin, and essential oils including its major component cinnamaldehyde (Molania et al. 2012) to probiotic yogurt can result in increasing numbers of live probiotic bacteria, and the antioxidant activity of the yogurt (Shori and Baba 2011). Cinnamon not only has antioxidant properties, but also can express antibacterial effect on some pathogens (Cardoso- Ugarte et al. 2016). Recent studies and research opinions might lead to the suggestion that the combined intake of probiotics, propolis and cinnamon via a novel functional food product could be a simple and tasty preventive approach to viral infections. Apart scientific evidence regard-ing antiviral and/or immunostimulating effects of the respective functional food product it is the first and foremost to determine potential interferences of the ingredients. Therefore, we aimed to study the impact of propolis and cinnamon on the microbiological, chemical, and sensory properties of probiotic yogurts developed as a novel functional food product.

## Materials and methods

### Materials

The probiotic yogurt preparation culture used in this study was obtained commercially from Maysa Gida, Adana (Turkey). Milk samples were taken from the supermarket (Turkey). Propolis used in this study was purchased from Sepe Natural Organic Products (Turkey). According to the manufacturer, each 10 mL liquid water-based propolis product contains 300 mg of Brazilian propolis (3.5 mg Gallic Acid and 2.1 mg Quercetin per mL product). Cinnamon was commercially obtained from Phytotherapy Center, Bezmialem Vakif University (Istanbul, Turkey). As reported in our previous study, the amounts of essential oils in powdered cinnamon are listed from high to low, respectively: cinnamaldehyde, alpha copaene, caryophyllene, eucalyptol and others (Günes-Bayir and Bilgin 2019). Peptone, which is used in microbiological analyzes, is commercially provided in powder form from Merck (Turkey). Distilled water was produced in our department laboratory at the Bezmialem Vakif University. MRS, MRS-CC (MRS-clindamycin-ciprofloxacin), M17 and TOS-MUP (Transgalactosylated oligosaccharidesmupirocin lithium salt) agars were purchased commercially from Merck (Turkey) and used for the detection of *Lactobacillus delbrueckii* subsp. *bulgaricus*, *Lactobacillus acidophilus*, *Streptococcus thermophilus*, and *Bifidobacterium animalis* ssp. *lactis*, respectively.

### Preparation of probiotic yogurt groups

**Fig. 1:**
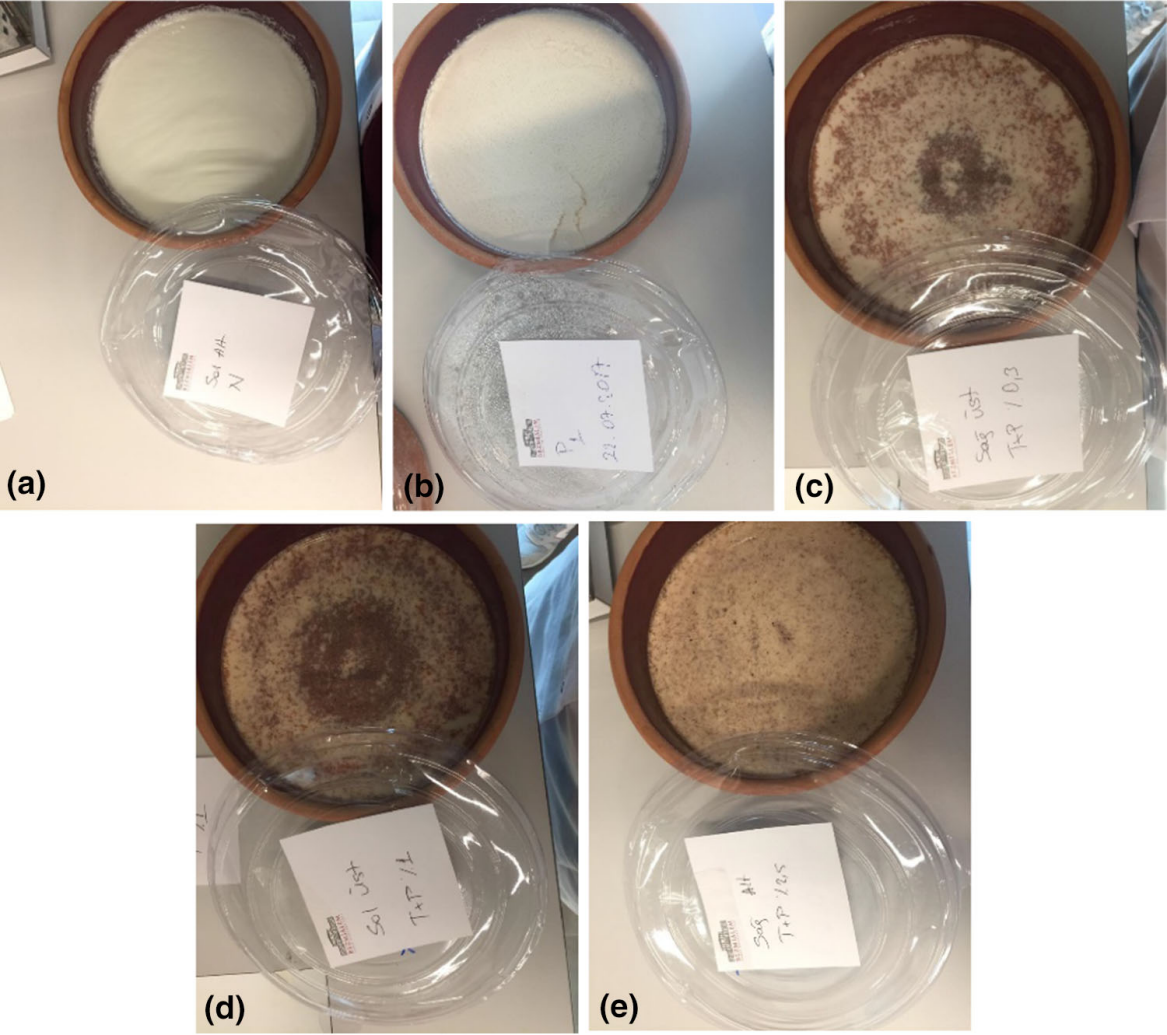
All experimental probiotic yogurt groups were presented as **a** yogurt without propolis and or cinnamon (control group); **b** yogurt with propolis (0.03%); **c** yogurt with propolis/cinnamon (0.03%/0.3%); **d** yogurt with propolis/cinnamon (0.03%/1%); **e** yogurt with propolis/cinnamon (0.03%/2.5%), respectively

Five-hundred mL pasteurized milk were heated to 42 °C. Fifty mL of heated milk were fermented with commercial purchased starter cultures. Then, the cinnamon was added in different concentrations (0.3%, 1%, and 2.5%), whereas the propolis was added at a concentration of 0.03% for the experimental yogurt groups (Fig. 1). Fifty mL mixture was then transferred into 450 mL of the previously heated milk (42 °C). After 6 h of further incubation, the yogurt was taken from the oven, and cooled at the room temperature. The cooled yogurt was placed in a refrigerator at + 4 °C for 24 h to ensure the maturation of yogurts. In addition, a yogurt was prepared with adding propolis (0.03%), and a yogurt was without propolis and cinnamon, which was used as the control. The preparation and analyzes of each experimental probiotic yogurt group was repeated three times.

### Microbiological analysis of probiotic yogurt groups

A peptone powder (0.1%) was added into 2 L of sterile distilled water to obtain peptone water. The prepared peptone water was sterilized using an autoclave (Nüve Materials Manufacturing Industry and Trade Co., Turkey). The microbiological analyzes of probiotic yogurts were performed as follows: Ten g of sample were taken and placed in a sterile stomacher® bag. Ninety mL sterile peptone water was added into the stomacher bag. This mixture was used as a starting material (10^–1^ dilution) for microbiological analysis of yogurts after being homogenized in stomacher (VWR, Italy). One mL sample of this starting material was added to a glass tubes containing 9 mL of peptone water, and mixed. One mL of this homogenized mixture was transferred into a second tube containing 9 mL of peptone water and mixed. After that 1 mL of the mixture was added into the third tube and mixed again. So, dilution series were made so that the concentrations would be from 10^–2^ to 10^–11^. These obtained dilutions were added to MRS, MRS-CC, M17, and TOS-MUP agars by taking 100 µl of mixture from each tube. MRS, MRS-CC, and TOS-MUP were incubated at 37 °C for 72 h in an incubator (BINDER, Germany) in anaerobic environment according to IDF/ISO (Süle et al. 2014). M17 agar was incubated in aerobic environment at 45 °C for 24 h. Agars were evaluated using a colony counter (Interscience, France). Plates containing 25–250 colonies were counted, and the results were expressed as colony-forming units per mL (cfu/mL).

### Chemical analyzes of probiotic yogurts

For the quantitative analysis of fat in yogurts, 5 mL of ammonia (Merck 28–30%) were mixed into 50 g of yogurt sample. Eleven mL of sample, 10 mL of sulfuric acid (Merck 90%, d = 1.82) and 1 mL of N-amyl alcohol (Merck 100%, d = 0.815) were added into the butyrometer, and centrifuged for 5 min (Funke Gerber Germany, T = 65 ºC, 1350 rpm). The fat values of the centrifuged butyrometers were measured. The same process was performed once more for control group (TS 1330) (Turkish Standards 2006).

Fat free dry matter analysis of yogurts were performed according to Turkish Standards (TS 1330) (2006). Petri dishes added with sea sand were dried in the oven. When these petri dishes cooled in desiccator, they were weighed in a precision weighing scale. Five grams of yogurt sample was added in order to incubate for 2 h at 103 ± 2 °C. After that, petri dishes were cooled in desiccator, and weighed in the precision weighing scale.

Quantitative analysis of protein content in yogurts were carried out by formol titration method (TS 1330) (2006). Fifty mL of yogurt sample was added into 50 mL of distilled water. After that, a 0.5 mL of 2% phenolphthalein solution (Merck, Turkey) and 2 mL of saturated potassium oxalate (Merck, Turkey) were mixed to be homogenized. After waiting 2 min, the mixture was titrated with 0.1 N NaOH (Merck, Turkey) to give a slightly pink color. Ten mL of formaldehyde (Merck, Turkey) were added, and mixed for 1 min. The value was measured by titration with NaOH.

For the determination of titratable acidity of yogurts, 10 mL of distilled water was added into 10 g of yogurt which was contained in the beaker. A 0.5 mL of phenolphthalein solution (1%) was added into the yogurt. This mixture was titrated with 0.1 M NAOH solution and remain unchanged pink color for about 30 s according to TS 1330 (Turkish Standards 2006). The pH value of yogurt samples was measured using a pH meter (Mettler-Toledo, Switzerland).

### Sensory analysis of yogurt groups

According to Turkish Standards 1330 (2006), the sensory evaluation of probiotic yogurts was performed by our study team of 8 researchers blindly. Five different yogurts (Fig. 1) were three time produced. Yogurt samples (*n* = 15) were presented to the panelists at the same time. According to TS 1330, yogurts were evaluated for their four characteristics: appearance, consistency, smell, and taste. Each characteristic is scored from 1 to 5 points. Each yogurt should get at least 4 points from each characteristic, and at least 16 points in total (TS 1330) (2006).

### Statistical analysis

A total of 15 produced probiotic yogurts were analyzed using SPSS 21.0 statistical package program. All data were tested using one-way ANOVA test and post hoc analysis of five different yogurts were performed by Tukey’s test. *p* < 0.05 was considered as statistical significance. The results of chemical analysis are demonstrated as mean ± standard deviation. The data obtained from sensory analysis were calculated for each group.

## Results

### Effects of propolis and cinnamon on microbiological properties of yogurts

**Fig. 2:**
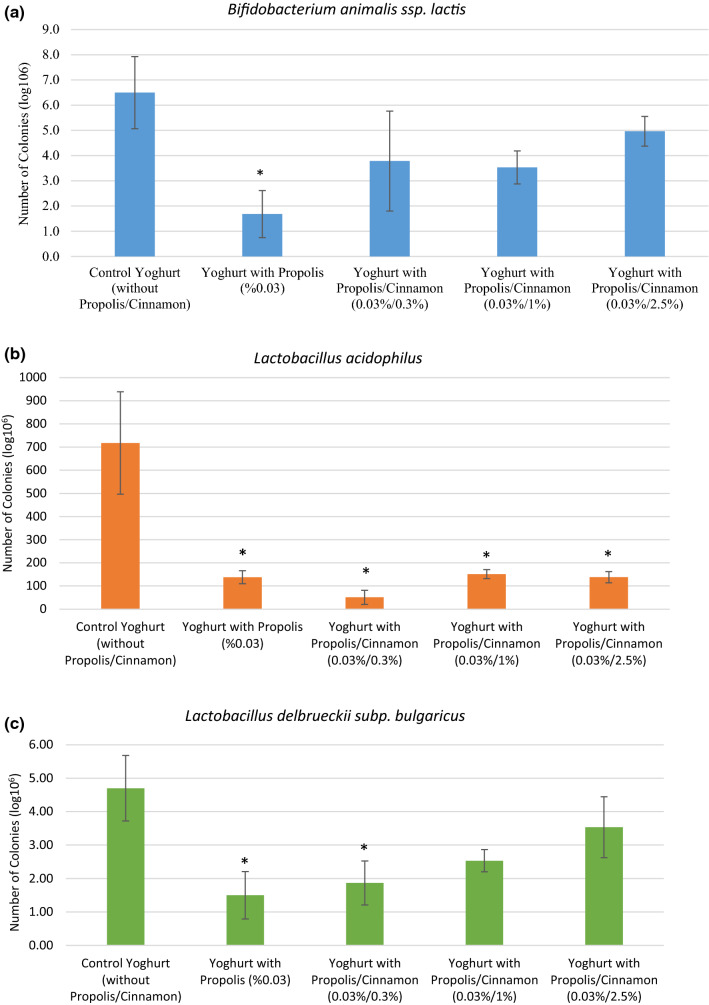

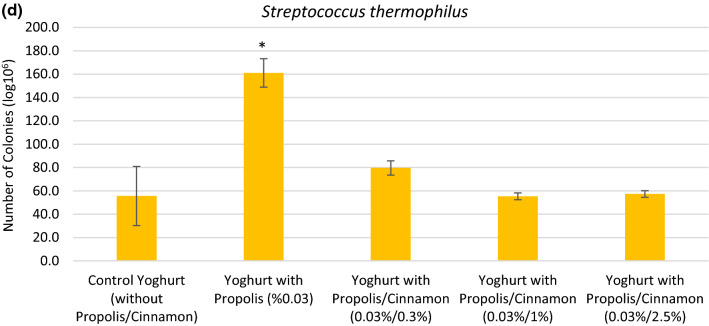
The bacteria counts in probiotic yogurt groups are determined as colony forming unit. **a** *Bifidobacterium animalis* ssp. *lactis*, **b** *Lactobacillus acidophilus* **c** *Lactobacillus delbrueckii* subp. *bulgaricus*, and **d** *Streptococcus thermophilus.* All values are expressed as the mean ± SD. * Significant differences were considered as p ≤ 0.05. SD: Standard deviation

The number of each bacterium was calculated, the results were compared among the experimental yogurt groups in Figs. 2a–d. Propolis had a statistically significant suppressive effect on *Bifidobacterium animalis* ssp. *lactis* in probiotic yogurt, (Fig. 2a.). Probiotic yogurts containing 0.03% propolis and 2.5% cinnamon showed higher *Bifidobacterium animalis* ssp. *lactis* numbers than other probiotic yogurts with propolis and cinnamon (0.03%/0.3% and 0.03%/1%). The number of colonies obtained from probiotic control yogurts were higher than those from yogurts containing cinnamon and propolis but these differences were not statistically significant except for yogurts with propolis alone. A significant effect on colonie numbers of *Lactobacillus acidophilus* was observed for all probiotic yogurts containing either propolis alone or in combination with cinnamon (*p* < 0.05) (Fig. 2b.). Propolis alone showed a statistically significant reducing effect on the number of *Lactobacillus delbrueckii* subsp. *bulgaricus* (*p* < 0.05) (Fig. 2c). However, this effect diminished in the presence of cinnamon in a concentration dependent manner to a none statistically significant decrease in numbers of *Lactobacillus delbrueckii* subsp. *bulgaricus* colonies in 1% cinnamon/0.03% propolis yogurts and 2.5% cinnamon/0.03% propolis yogurts, respectively. In produced probiotic yogurts, propolis showed a statistically significant increasing effect on the number of *Streptococcus thermophilus* colonies (*p* < 0.001). Products containing additional cinnamon showed an evident but not statistically signifcant increase in colonie numbers of *Streptococcus thermophilus* compared to controls which was below the effect of propolis alone (Fig. 2d).

### Effects of propolis and cinnamon on chemical properties of yogurts

For the determination of fat, fat free dry matter, protein, titration acidity and pH values of yogurts, chemical analyzes were carried out (Table 1). The findings revealed that the amount of fat decreases slightly in the presence of propolis and cinnamon, whereas the amount of fat free dry matters increased. Propolis as well as cinnamon did not show any effect on the protein content of yogurts. The results of titration acidity showed an increase in samples containing propolis alone, whereas all yogurts containing cinnamon in combination with propolis revealed a concentration dependent decrease in titration acidity. Yogurts with highest cinnamon content offered the lowest value for the titration acidity (*p* < 0.05). These results were in correlation with the determined pH value of the yogurt groups. Yogurt with propolis alone showed a slightly lowered pH value, whereas yogurts with highest cinnamon content had the significantly highest pH value (*p* < 0.05).

**Table 1:** Chemical analysis of experimental probiotic Yogurt groups including fat, fat-free dry matter, protein and titratable acidity in percentages, and pH values are demonstrated as mean ± SD. SD: Standard deviation. ^a^*p* < 0.05

	Control Yogurt (without Propolis/ Cinnamon)	Yogurt with Propolis (0.03%)	Yogurt with Propolis/ Cinnamon (0.03%/0.3%)	Yogurt with Propolis/ Cinnamon (0.03%/1%)	Yogurt with Propolis/ Cinnamon (0.03%/ 2.5%)
Fat Matter (m/v %)	3.3 ± 0.01	3.19 ± 0.01	3.15 ± 0.63	3.2 ± 0.01	3.15 ± 0.63
Fat Free Dry Matter (m/m %)	8.15 ± 0.1	8.18 ± 0.15	8.04 ± 0.41	8.25 ± 0.36	9.52 ± 0.03^a^
Protein (m/m%)	3.54 ± 0.02	3.54 ± 0.01	3.53 ± 0.025	3.53 ± 0.2	3.53 ± 0.25
Titratable Acidity (m/m %)	0.91 ± 0.06	1.02 ± 0.002	0.85 ± 0.005	0.85 ± 0.027	0.74 ± 0.01^a^
pH value	3.98 ± 0.092	3.91 ± 0.01	4.06 ± 0.078	4.05 ± 0.078	4.23 ± 0.11^a^

### Effects of propolis and cinnamon on sensory properties of yogurts

The results of sensory analyzes of yogurts are presented in Table 2. The control group became the highest total points as 160. The probiotic yogurt with propolis (0.03%) and cinnamon (2.5%) gained the highest number of points (154) compared to control, followed by yogurts conaining 0.03% propolis alone or in combination with 0.3% cinnamon (153 points each). Yogurt with propolis (0.03%) and cinnamon (1%) gained the lowest number of points (148).

**Table 2:** The sensory analysis of each probiotic yogurt group is presented according to Turkish Standards (TS 1330)

	Control Yogurt (without Propolis/ Cinnamon)	Yogurt with Propolis (0.03%)	Yogurt with Propolis/ Cinnamon (0.03%/0.3%)	Yogurt with Propolis/ Cinnamon (0.03%/1%)	Yogurt with Propolis/ Cinnamon (0.03%/ 2.5%)
Appearance	40	39	39	40	37
Texture	40	40	40	40	40
Odour	40	37	37	33	38
Taste	40	37	37	34	39
Total	160	153	153	148	154

## Discussion

In the course of global research on COVID-19 and related topics such as managing immune responses and various disease conditions, preventive approaches to SARS-CoV-2 infections are of utmost interest. Studies focusing on antiinflammatory, immunoregulatory as well as antiviral activity include pharmacological and nutraceutical agents. Better knowledge of the virus and its enzymes will aid the development of specific antivirals as better knowledge of how host genetic variants may contribute to severity of COVID-19 infection will help to identify patients at high-risk for severe COVID-19 (Lima et al. 2020). At present treatment is essentially supportive and hence preventive measures of highest importance. On the other hand, latest data from clinical trials lead to evidence that consumption of fermented dairy products are highly important if dosages are determined (Polamarasetti and Martirosyan 2020). Additionally, further clinical studies should be providing support for their use against COVID-19 infection, but also bioactive compounds and functional foods should be existing in a prophylactic nutrition plan for humans. In recent times, there has been an increased interest to propolis, cinnamon and probiotics, since potent activities and effects have been demonstrated such as inhibition of the attachment of SARS-CoV-2 to ACE2 for propolis (Berretta et al. 2020) or inhibition of the S1 subunit of spike proteins of SARS-CoV-2 which is involved in the interaction with ACE2 receptors for cinnamon components (Kulkarni et al. 2020). Positive impact on host immune functions is documented not only for propolis and cinnamon but also for probiotics (Babaei et al. 2016; Cardoso-Ugarte et al. 2016; Alkhatib 2020; Hu et al. 2021; Singh and Rao 2021). Thus, it is attractive to speculate that combined intake of probiotics, propolis and cinnamon via a novel functional food product could be an eligible approach in helping prevent severe SARS-CoV-2 infections. Viral infections are characterized by a weak immune system and insufficient present of micronutrients (vitamins and minerals) in human body (Alkhatib 2020). Consuming adequate diet and supplementation of functional foods the immune system can improve. In the process of developing such functional food product it is utmost important to determine potential interferences of the ingredients. In our study, we used *Bifidobacterium animalis* ssp. *lactis*, *Lactobacillus acidophilus*, *Streptococcus thermophilus*, and *Lactobacillus delbrueckii* subsp. *bulgaricus* as microorganisms to produce probiotic yogurts and examined the effects of propolis and cinnamon at different concentrations on the viability of the bacteria. On the other hand, the internal use of propolis according to EFSA report 0.7–1.3 g/day and the safe usage limit is 2 g/kg/day propolis. Use of propolis extract 1 or 3 times a day at least 250 mg/day for children, for adults 750 mg/day (EFSA 2018). Yogurt added propolis extract in different proportions (0.01, 0.03, 0.10, and 0.20%) in a study, it was found that the addition of propolis increased the antioxidant activity of yogurt and slowed down its microbial growth (Güney 2016). In another study, the different proportions of propolis (0.1, 0.2, 0.3, 0.4, and 0.5%) affected physical and chemical propeties of ice creams, but sensory properties are somewhat negative (Mehmetoğlu et al. 2019). According to all these literature, we decided the use of 0.03% propolis in our study, which can be obtained commercially.

In recent years, the antibacterial and antifungal effects of cinnamon have been studied, and discussions are still ongoing (Choi et al. 2016). It has been reported that cinnamon could be used for antimicrobial effects against main pathogens causing foodborne diseases such as *Staphylococcus aureus* in milk and yogurt or *Salmonella typhimurium*, *Listeria monocytogenes, Escherichia coli, Bacillus cereus*, and *Pseudomonas aeruginosa* (Nematollahi et al. 2020). In this sense cinammon could also offer beneficial effects in the novel functional food product but does it also alter the probiotic bacteria *Bifidobacterium animalis* ssp. *lactis, Lactobacillus acidophilus, Lactobacillus delbrueckii* subsp. *bulgaricus*, and *Streptococcus thermophilus* to an extent rendering them ineffectiv in the probiotic yogurt produced? To answer this question microbiological analyzes were performed. Cinnamon at concentration up to 2.5% had no statistically negative effect on numbers of *Bifidobacterium animalis* ssp. *lactis*, a probiotic for which Miller et al.(2017) reported that daily consumption enhances NK cell and PMN function in healthy elderly adults. It turned out that cinnamon was even able to reverse negative effects of propolis, which caused a statistically significant decrease in colonie numbers when added alone. The same effect was seen for *Lactobacillus delbrueckii* subsp. *bulgaricus* whereas a reverse effect was detected for *Streptococcus thermophilus*. For the latter, propolis had a strong and statistically significant positive effect on growth which was abolished by cinnamon in a dose-dependent manner. At the highest cinnamon concentration of 2.5%, the propolis effect was totally abolished and values for *Streptococcus thermophilus* were back to control. Propolis with or without cinnamon had statistically negative effects on the number of colonies of *Lactobacillus acidophilus*. Unlike detected for *Lactobacillus delbrueckii* subsp. *bulgaricus*, cinnamon was not able to reverse the negative effect of propolis on bacterial growth for this member of the *Lactobacillaceae* family. This observation is not surprising, since it has been shown that cinnamon negatively affects the growth of *Lactobacillus acidophilus* itself (Günes-Bayir and Bilgin 2019).

In general, various factors such as strain variation, acid accumulation, level of dissolved oxygen and hydrogen peroxide influence the viability of probiotics in yogurts (Talwalkar and Kailassapathy 2003). The medical activity of probiotic food products depends on the number of active cells or the total number of viable cells per mL or gram of the product (Nigam 2018). Even though various effects of propolis and cinnamon were found on the number of probiotic microorganisms under investigation, all samples met the standard of recommended level of ≥ 10^6^ viable cells/g of a product (Turkish Food Codex on Labeling and Informing Consumers Regulation/Supplement 15).

Chemical analyzes of yogurts revealed a decline of fat matter in the presence of propolis and/or cinnamon which did not correlate with increasing cinnamon concentrations. This is in accordance to findings in yogurt from goat milk where the fat ratio decreased as the rate of cinnamon extract added increased (Lindasari 2013). On the other hand, fat free dry matter increased an effects that has been shown for cinnamon in a previous study (Günes-Bayir and Bilgin 2019) and was correlated to increasing cinnamon levels. The amount of protein did not change in the presence of either propolis or cinnamon. This result may be related to the very low protein content of propolis and cinnamon. It was reported that no significant differences in the values of titration acidity and pH occured when cinnamon was added to probiotic yogurts from cow and camel milk (Sori and Baba 2011). Choi et al. (2016) published results similar to our study, the pH value was decreased by 0.19% in yogurts prepared with the ethanol extract of cinnamon, while the value for the titration acidity was increased by 0.28%. In our study yogurts with the highest cinnamon concentration (2.5%) and propolis (0.03%) showed the lowest titration acidity and the highest pH values. This trend was also evident with lower cinnamon concentrations. However, there was no difference between samples containing 0.3% and 1% cinnamon and propolis. In contrast to these results, in our previous study, the increase in pH values and the decrease in titration acidity of yogurts were depending on increased concentration of cinnamon (Günes-Bayir and Bilgin 2019). The findings in the present study depend on the increase of titration acidity by propolis which also led to a lesser increase of pH value.

It was revealed that an association exists between the acceptability and the acidity of yogurts (Çakmakçi et al. 2012). Additionally, the results of sensory evaluation of probiotic yogurt added the lowest cinnamon concentration (0.3%) was found to be similar for probiotic yogurt without cinnamon (Günes-Bayir and Bilgin 2019). In the present study, the probiotic yogurt without propolis and cinnamon had the highest number of points regarding the sensory evaluation. On the other hand, yogurts containing propolis and cinnamon (0.3% and 1%) demonstrated the same values for pH and titration acidity. However, total points as the result of sensory analysis were not the same. Briefly, propolis and cinnamon affected negatively on sensory properties of probiotic yogurt.

In conclusion, the number of probiotic bacteria was found to be more than 1.0 × 10^6^ cfu per gram in all produced probiotic yogurts. The addition of cinnamon into probiotic yogurt with propolis can affect the microbial and chemical qualities of probiotic yogurts with propolis but may have limited effect on their sensory properties. Further investigations can help to improve on the taste of this functional food product to ensure palatability. In a further step in vitro, in vivo, and clinical studies regarding antiviral and/or immunostimulating effects of the respective functional food product will be conducted.

## Data Availability

The data that support the findings of this study are available from the corresponding author, upon reasonable request.

## Abbreviations

ACE2: Angiotensin-converting enzyme 2
CFU: Colony forming unit
IDF: International Dairy Federation
ISO: International Organization for Standardization
TS: Turkish Standards
